# Community perceptions and factors influencing utilization of health services in Uganda

**DOI:** 10.1186/1475-9276-8-25

**Published:** 2009-07-14

**Authors:** Solome K Bakeera, Sarah P Wamala, Sandro Galea, Andrew State, Stefan Peterson, George W Pariyo

**Affiliations:** 1 Department of Policy and Planning, Makerere University School of Public Health, Kampala Divison of Social Medicine, Uganda; 2 Department of Public Health Sciences, Karolinska Institutet, Stockholm, Sweden; 3 Division of Social Medicine, Department of Public Health Sciences, Karolinska Institutet, Stockholm, Sweden; 4 Center for Social Epidemiology and Population Health, University of Michigan, Ann Arbor, MI, USA; 5 Faculty of Social Sciences, Makerere University, Kampala, Uganda; 6 Divison of International Health, Department of Public Health Sciences, Karolinska Institutet, Stockholm, Sweden; 7 Department of Policy and Planning, Makerere University School of Public Health, Kampala, Uganda; 8 Current: Swedish National Institute of Public Health, Ostersund and Karolinska Institutet, Stockholm, Sweden

## Abstract

**Background:**

Healthcare utilization has particular relevance as a public health and development issue. Unlike material and human capital, there is little empirical evidence on the utility of social resources in overcoming barriers to healthcare utilization in a developing country context. We sought to assess the relevance of social resources in overcoming barriers to healthcare utilization.

**Study Objective:**

To explore community perceptions among three different wealth categories on factors influencing healthcare utilization in Eastern Uganda.

**Methods:**

We used a qualitative study design using Focus Group Discussions (FGD) to conduct the study. Community meetings were initially held to identify FGD participants in the different wealth categories, ('least poor', 'medium' and 'poorest') using poverty ranking based on ownership of assets and income sources. Nine FGDs from three homogenous wealth categories were conducted. Data from the FGDs was analyzed using content analysis revealing common barriers as well as facilitating factors for healthcare service utilization by wealth categories. The Health Access Livelihood Framework was used to examine and interpret the findings.

**Results:**

Barriers to healthcare utilization exist for all the wealth categories along three different axes including: the health seeking process; health services delivery; and the ownership of livelihood assets. Income source, transport ownership, and health literacy were reported as centrally useful in overcoming some barriers to healthcare utilization for the 'least poor' and 'poor' wealth categories. The 'poorest' wealth category was keen to utilize free public health services. Conversely, there are perceptions that public health facilities were perceived to offer low quality care with chronic gaps such as shortages of essential supplies. In addition to individual material resources and the availability of free public healthcare services, social resources are perceived as important in overcoming utilization barriers. However, there are indications that having access to social resources may compensate for the lack of material resources in relation to use of health care services mainly for the least poor wealth category.

**Conclusion:**

The differential patterning of social resources may explain or contribute to the persisting inequities in health care utilization. Additional research using quantitative analytical methods is needed to test the robustness of the contribution of social resources to the utilization of and access to healthcare services.

## Introduction

Utilization of healthcare services is an important determinant of health [1-3], and has particular relevance as a public health and development issue in low income countries [4]. In fact, utilization of healthcare services for the most vulnerable and underprivileged populations has been recommended by the World Health Organization as a basic primary healthcare concept [5]. It has been suggested that healthcare should be universally accessible without barriers based on affordability, physical accessibility, or acceptability of services [4,6]. Accordingly, increased use of health services is a major target in many developing countries [7].

In Uganda, the Health Sector Strategic Plan (HSSP) endorsed by the Government and Development Partners prioritizes key actions to attain agreed upon sector targets. The first (2000–2005) and second (2006–2010) HSSP periods have focused on ensuring universal access to a minimum healthcare package of services^i^. Universal access is a core strategy for achieving increased healthcare utilization and reducing the disparities therein [8,9]. In general, utilization of health services has improved over both HSSP periods [10,11]. However, other studies conducted for the same period suggest that inequities in the use of health services persisted. For instance, the analysis of the Uganda National Household Survey Data [Uganda National Household Survey data 1997/98, 1999/00 and 2002/03] shows that the reduction in those reported not to seek care was more for the richest (5.3%) as compared to the lowest quintile (2.3%), whilst there was an increase in reported 'no care' for those in the second (3%), third (1.5%) and fourth (3.2%) quintiles respectively. Other inequalities observed were that children in the < 5 years age category had the highest reported percentage of 'no care' during 1997/8 to 2002/03. More women than men reported receiving no care and up to 50% of the categories that make up 'no care' are in the lowest and second lowest quintile of the study population [12].

The interdependent factors that determine utilization of health services are aptly described in a Health Access Livelihood Framework [4]. They are related to the health seeking process, the nature and organization of health services and to the access of livelihood assets [4]. In the process of seeking health care, people will use services if they find them to be acceptable. How acceptable services are is related to the nature and organization of services which includes their availability, accessibility, affordability and adequacy; this encompasses the health services approach [6,13]. Livelihood assets include financial capital, physical capital, natural capital, human capital, and social capital. Financial capital comprises cash and credit whilst physical capital includes the infrastructure, equipment and means of transport. Natural capital refers to land, water and livestock [4]. In this study we broadly refer to financial, physical and natural capital as material resources. The linear relationship between material resources and healthcare utilization is widely documented [14-18]. Human capital refers to local knowledge, education and skills [4]. Studies in both high and low income countries link higher education status with more use of health care services [19,20]. The relationship between social resources and healthcare utilization is widely documented in a high-income country setting but has rarely been empirically investigated in low-income countries [21-29]. Consistent with others, we think that social resources are a feature of social capital and mean the term to refer to the diverse resources embedded in social networks [30]. Social networks are integral to the definition of social capital and refer to associational activity between two or more persons [31]. A study done in West Africa by Ayé et al [32] did show that in spite of poverty, many poor people in Ivory Coast were able to access expensive modern health care services due to availability of social networks. This is because in many African countries illness is often regarded as a social phenomenon, as it limits participation in community life. Therefore, treatment of a sick person becomes a community action tied to the collective systems of life including participation of family, friends and acquaintances. This highlights the importance of social resources in mobilization of resources that enable utilization of health care services. Additionally, a 15-country African study shows that borrowing from friends was one of the coping strategies employed to finance hospital care. It however excludes the poorest that were unable to seek care [33].

The aim of this study was to explore community perceptions about barriers and facilitating factors influencing healthcare utilization in Uganda amongst different wealth status groups. This was in order to assess the relevance of social resources in overcoming barriers to healthcare utilization amongst different wealth status groups. Existing studies in Uganda that have investigated patterns of healthcare utilization have not considered the role of social resources [34-37]. Those that have, do not explicitly examine the role of social resources within different wealth categories [20,38].

## Methods

### Study Area

A qualitative study design explored factors influencing healthcare utilization. The study was done between December 2007 and March 2008 in the neighborhood of the Iganga/Mayuge Demographic Surveillance Site (DSS). Three villages (Nawangisa village, Bukona-Kakongoka village (a/b) and Namundudi) of Kigulu South Health Sub-District (HSD) are included. These villages are designated for exploratory research leading up to further studies in the DSS. The HSD like the rest of Iganga district has a 4-tier healthcare system, with increasing scope and complexity of services offered from community healthcare workers at village level up to Iganga general hospital which provides overall leadership for health services within the HSD.

Typically, the area is characterized by low service provision with inadequate human and health care financing resources. The total number of registered facilities include: 18 Health Centre (HC) II (7 public; 8 private-not-for-profit; 3 private); 4 HC III (all public); 1 general hospital. Most of the government/public-owned HC II fulfill the minimum staffing norms but none of the HC III have the full complement of staff. In the whole district including Kigulu health sub-district, only 54% of approved posts are filled with professional workers. Sixty eight percent of the district population lives within a 5 km radius of either a public or private not-for-profit health facility compared to the HSSP II target of 75%. The realized health sector non-wage recurrent budget per capita in the whole district was 4,713 UShs (USD 2.80) in 2006/07. Of this UShs 386 (20 US ct) per capita was allocated to Kigulu South health sub-district to support activities at lower level facilities. The general hospital had a budget of Ushs 505 (30 US ct) per capita. The per capita utilization of outpatient department services dropped from 58% in 2005/06 to only 40% in 2006/07 [39].

#### Planning Focus Group Discussions (FGDs)

##### a) Identification and recruitment of FGD participants

In the absence of socioeconomic status indices for households where the study was conducted, a modification of poverty ranking as described in Theis and Grady [40] was used to classify different wealth categories in each of the villages. We held community meetings in each of the three villages which identified criteria for categorizing village members into three different wealth categories (See Appendix 1 and Table 1).

**Table 1:** Summary of wealth ranking exercise

**Socioeconomic characteristic**	***'Abagaiga' *– The least poor**	***'Abafuni' *– (The Medium wealth category)**	**Abaavu – The poorest**
**Income (financial capital)**	Income generating activity e.g. shop or other business with capitalization from 400,000 – 1 million UGX	Low earning, unable to save	- Unable to raise small amounts (2,000 Ushs) in a crisis e.g. police bond or cannot have on them 1000 Ushs - Lacks a job that can bring in daily income - May have small amount of capital from 20,000 – 30,000 Ushs

**Assets** **(physical and natural capital)**	Means of transport – car or motorcycle; House – building materials – bricks, plastered walls, iron sheets, glass windows; state of maintenance and surrounding compound as important as building materials; Farmed land up to 5 acres	Cows, goats, hens with value up to 100,000 Ushs	Doesn't have anything Squalid home Poor beddings No material things to sell

**Occupation**	Self-employed in some business	Primary school teachers, petty traders	Does not work due to advanced age, illness, irresponsible social behavior, appearance (cannot obtain employment) Employment of casual nature

**Ability to survive**	Can obtain all needs in a timely manner without straining themselves	Can solve problems of magnitude up to 100,000	- Lives hand to mouth - Cannot survive without borrowing or asking for assistance - Survives on social/community support e.g. needs to borrow or ask for help to obtain certain elements for survival - Usually lacks close family relatives

The village chairperson together with the village mobilizer who acted as guide used these wealth status criteria to select focus group participants who fitted each wealth category. We believe that the guide knew village members personally since he lives with them and is regularly engaged in mobilization activities that bring him into close contact with households. Additional criteria for selection included the subjective assessment by the village guide for the participant's potential to discuss openly. Selected participants were approached to participate in the Focus Group Discussions (FGDs) the following day at a venue removed from the general public. Most of the venues were village meeting places that usually shielded participants from bad weather (in case of rain or intense heat from the sun) and this usually followed consultation with selected participants for its appropriateness.

##### b) Establishing the number of focus groups

We planned to have three FGD in each of the villages giving us a total of nine FGD, with the option of adjusting the number should information saturation be arrived at with less or more. Homogeneity for each group was on the basis of wealth categories determined by the community identified criteria. Other criteria considered in the selection were sex, age category and disability status (see table 2). As much as possible, members who had participated in the community meeting were excluded from the FGD. This was in order to optimize information triangulation with regard to aspects of wealth ranking. However, owing to the enthusiasm at the community meeting the previous day, uninvited community members did present themselves at the FGD venue particularly for Nawangisa village, resulting in slightly larger than planned numbers. This decision to concede to slightly more numbers than the recommended 8–10 was based on the need to maintain community goodwill since we had other ongoing data collection. For the least poor and medium wealth category, uninvited participants who showed up and did not fit the criteria were requested by the village chairperson to leave. In the poorest wealth category, uninvited members in most cases fitted the criteria and were only requested to leave when the number exceeded 15. From the experience in Nawangisa where it proved relatively difficult to control numbers, meetings in Namundudi and Kakongoka took place in the compound of a private home, either on a verandah attached to the house or a separate resting shade. Each of the FGD lasted about one and a half hours. The numbers in each of the focus group discussions are summarized in table 3. In all there were a total of 88 participants.

**Table 2:** Characteristics of participants at focus group discussions in Nawangisa village

**Category**	**Total number of participants**	**Men**	**Women**	**Occupations of participants**	**Other observations**
Least Poor "*Abagaiga*"	8	5	3	Mulimi (1 acre of rice, 1 acre of maize), produce buyer; Affiliate manager for habitat for humanity; health worker/nurse; peasant animal farmer with 1–2 cows	Observation – the rich were contacting each other with mobile phones to come for the meeting

Middle wealth category *"Abafuni"*	15	11	4	Mainly peasant farmers who also trade in farm produce, rear chicken, goats for trade on a small scale, primary school teachers	

Poorest *"Abaavu"*	12	11	1	Types of occupation present – peasant crop farmers (ages, 23, 47, 55 yrs), local security man, 70 year old – can't dig anymore, another too old to work since 1992 has been sickly, not able to work, another urethral obstruction, difficult vision, no savings; no energy to dig has hernia, backache, poor vision 62 year old, 48 year old – no job, casual laborer.	The LC 1 chairman was present at this meeting

**Table 3:** Numbers in each of the Focus Group Discussions by village, wealth category and sex

**Village name**	**Least Poor**	**Medium wealth category**	**Poorest**	**Total**
Nawangisa	**8**	**15**	**12**	**35**
Men	5	11	11	27
Women	3	4	1	8

Kakongoka	**9**	**8**	**8**	25
Men	5	4	4	13
Women	4	4	4	12

Namundudi	**7**	**12**	**9**	28
Men	5	6	5	16
Women	2	6	4	12

Total	24	35	29	88

##### c) Scheduling the focus group

In consultation with the local leaders and observation of the socioeconomic activities, FGD meetings were usually conducted in the afternoons to allow participants tend to their farms and business activities in the morning. This allowed ample time to the research team to conduct village transect walks and get familiar with the settings.

##### d) Implementing the focus group discussions

The FGD method is particularly suited to capturing new information [41] on the participants' lived accounts of events at both the individual and group levels. The FGDs explored the following themes – healthcare utilization, barriers and facilitating factors (See Appendix 2). All meetings, including the FGDs were conducted in Lusoga which is the local language. They were facilitated by a social scientist with proficiency in Lusoga and good knowledge of the culture. With permission from the participants, all discussions were digitally recorded and backed up by field notes by a trained research assistant and the health specialist (SKB) who has worked professionally with the Uganda health systems in both the public and private sectors. Field debriefs were held on the same day after the meetings to check for consistency and completeness of recording, as well as any unique and emerging issues the research team encountered. The social scientist then transcribed all voice recordings while referring to the hand-written notes.

### Analyses

The Health Access Livelihood Framework was used to examine and interpret the findings. This framework described by Obrist et al provides a schema within which we can consider health service and health seeking-approaches in light of potential livelihood assets and actions [4]. The health service approach is related to the nature and organization of health services and focuses on the availability, affordability and adequacy of services as they influence utilization. The health-seeking approach is related to acceptability of services and examines why, when and how individuals and communities seek access to healthcare services. The livelihood approach in this case was concerned with the assets needed to facilitate use of health services. The assets included in the livelihood approach included financial, social, human, natural and physical capital. These three approaches are interdependent and together influence the outcome of health care utilization [4]. Given that these approaches are interdependent, it is logical to assume that the presence of social capital improves utilization of health services by compensating for the lack of human capital and material resources among the poorest. This logic is motivated by current evidence in the literature [20,32,33,42].

Qualitative content analysis was done using open code version 2.2. Relevant statements in the transcripts were located and meanings were assigned to them. The meanings were synthesized into common themes within the Health Access Livelihood Framework.

### Ethical approval

The Higher Degrees Research and Ethics Committee of Makerere reviewed the study and recommended ethical approval (2006/HD20/4824U) which was granted by the Uganda National Council of Science and Technology. Written informed consent was sought from the District Health team, management of the Iganga-Mayuge Demographic Surveillance Site and the Local Council of the villages. Additionally, verbal consent was sought from all participants of the community meetings and focus group discussions. Soft drinks were provided at all meetings and FGDs.

## Results

Barriers and facilitating factors that were identified are grouped according to the thematic areas [43] in the Health Access Livelihood Framework [4]. These are described in the sections below:

### Barriers to healthcare services' utilization in the health seeking process

Service acceptability depended on the health worker attitudes and practices. An example of poor attitudes and practices is illustrated by the following quote:

*'The health workers treat us badly like we are not human beings, they may not even be bothered if someone dies compared with the traditional birth attendants who treat people humanely. In hospital they slap you and say "to avoid disturbances let's do the caesarian operation". This brings fear and skepticism in using the service. If you have grown up in poverty you may look older than you actually are and they will abuse you and say that "look that old woman who has come to give birth"' *(FGD Medium wealth category, Nawangisa).

Another practice that was noted to be unacceptable was perceived gender discrimination.

*'A customer is king *(but) *in the hospital women are mistreated because they may not have money. The men always have 'something" *(FGD, Least Poor, Nawangisa).

Local illness and treatment perceptions as well as fear and stigma were also identified as barriers in the health seeking process. For instance, it was recognized that illness could be acute and serious requiring attention depending on the perceived urgency. Conditions that were identified as needing conventional care include *'vomiting in children, convulsions, "pressure" *(hypertension)*, "ulcers" *(chronic epigastric pain)*, dental problems, cough and chest pain, epilepsy, diabetes, malaria, and measles'*. And yet, the perception that traditional care or faith healing was the norm for some conditions might deter conventional healthcare utilization for all wealth categories. This is illustrated in the quotes below:

*' Artificial medicine for "nawawa" *[condition where child presents with chills and cold spells]*, "eyabwe" *[convulsions]*, "syphilis" and sexually transmitted diseases is inferior to local medicines.' *(FGD Least poor, Kakongoka).

*'Some types of balokole (born-again Christians) just pray for conditions like fever, snake bite, diarrhea, serious fall from a tree' *(FGD Poorest, Nawangisa).

Related to the local illness perceptions was the lack of trust in the usefulness of certain interventions. This in some cases could deter the adoption of preventive actions:

*'It's not easy to prevent malaria because even if you say you want to buy a mosquito net, you won't put it on when you are outside of the home conversing' *(FGD Poorest, Namundudi).

Fear and stigma as barriers to use of some health services are illustrated in the quote below:

*'There are illnesses that we don't take to health units where we have someone known to us. Like HIV/AIDS, here we just go to witch doctors. In fact, we don't even want to know that it's AIDS. We prefer to be told that it is witchcraft. People fear to give advice when they see signs of AIDS. They are afraid because that would be offending the sick, people shall ask you how you know ... how you come to imagine that it's HIV. When you mention testing to them, they will shun you and never want to talk to you again' *(FGD Medium wealth category, Namundudi).

Steps in health seeking behavior could create delay in accessing appropriate treatment as illustrated in the quote below:

*'For us when you feel ill, you go to the drug shop and explain your pains to the attendant, who chooses the drugs. When things do not work out, you go to the private clinic. The clinic nurse is more technical than the drug shop attendant who when defeated may refer you to a health centre and in case the condition is worse you are taken to Nakavule Iganga Hospital' *(FGD Least Poor, Kakongoka).

Once illness was recognized and a decision made to seek care, not knowing where a service was provided led to choices of alternative or 'no-care'.

*'For us once you have AIDS, you just go to witch doctors otherwise we don't have places to go for treatment. Once you have HIV, you just wait to die' *(FGD Medium wealth category, Kakongoka).

### Health service factors as determinants of utilization

Determinants of utilization in this approach were related to whether services were available, adequate, acceptable or affordable.

#### Availability of services

Availability of services was a perception translated to mean that services were within reasonable physical reach. The poorest wealth category identified the availability of free public care as enabling to the use of both preventive and curative services:

*'If there isn't any money in the home we go to the health centre since we can sometimes get free treatment' *(FGD Poorest, Kakongoka).

The proximity of local private clinics was also considered essential as first aid points:

*'Our local health care options are near – some illnesses need first aid. For example acute illness such as convulsions, if a child has convulsions, we go to drug shops and private clinics because they are the nearest and also because we may not have transport to get to another facility' *(FGD Medium wealth category, Nawangisa).

The presence of community medicine distributors was also reported as facilitating use of conventional health care but these were considered unreliable:

*Community medicine distributors make it easy for us to access care but these are unreliable *(FGD Poorest, Namundudi).

#### Adequacy of services

The adequacy of services was judged in light of perceived quality, the way services were organized, and the availability of commodities. The inadequacy of health services was noted for preventive and curative care as well as at the different levels. For instance, the distribution of free commodities enabled use of preventive actions such as condom use but this was inadequate to cater for existing demand:

*'There is no way to get free condoms, maybe when there is an immunization outreach but we buy most of the time' *(FGD Poorest, Namundudi).

At the public health centre II, participants decried the approach of health care providers, the lack of supplies and equipment. There was also a sense of inconvenience in the process of obtaining care.

*'The health centre works only for twelve hours, there are always stock-outs, and health personnel are very rude and tough. No ambulance, poor referral system. Sometimes they delay to refer you to the hospital and may hold you only to tell you after a long time for example 6.00 pm when you can't do much. There is only one bed for maternity, few staff, they always insist to go by the book, may require of you unnecessary details and credentials etc.' *(FGD Least poor, Kakongoka).

There was also a lack of trust in the health worker qualifications particularly in the local private clinics and hence the efficacy of the treatment given.

*In our *(private) *clinics, we do not know the qualifications of our health workers. You can't ask them where they obtained their qualifications from, so long as they give you some treatment. For some conditions like severe anemia – these local health services cannot be useful yet you cannot afford referral. When you have fever you go and pay 200/- shillings worth of medication whatever it is. It could be chloroquine mixed with Aspirin^® ^we are never sure, whether it helps or not is another matter. You can't be sure that what you are getting is effective but we have no other option' *(FGD Poorest, Nawangisa). At the government hospital the lack of supplies compromised perceived adequacy of services even for the least poor as they found it unrealistic to purchase medicines from without late at night.

*Government hospitals have no supplies. There is a lack of medicines in government hospitals. You are sent to buy supplies at 2.00 am. Where do you buy supplies at 2.00 am ... so why bother to rush to a big hospital?' *(Least Poor, Nawangisa).

#### Affordability

High cost was identified as a barrier to the adoption of certain preventive actions such as use of mosquito nets and condoms. High cost was also a barrier to treatment of both acute and chronic conditions. An example of high cost as a barrier to use is demonstrated in the following quote:

*'Treatment of chronic diseases like AIDS, "ulcers" and "pressure" is expensive in terms of treatment and transport. It's not easy in fact someone can even die' *(FGD Medium wealth category, Namundudi).

In addition, although public services are supposed to be free of charge, demand for unofficial fees presents a barrier to use particularly for the poor:

*"There is someone who was taken by family members to the *(public) *hospital but even then he was brought back because they could not pay bribes to the medics" *(FGD, Least Poor, Namundudi).

### Livelihood assets as a determinant of use

Ownership of material, human and social resources that were reported as determinants of health care service use.

#### Material resources

Material resources that were related to use of services include financial, physical and natural capital. Financial capital in terms of cash and/or credit was identified as an important factor for use of services. Physical capital in terms of ownership of means of transport also facilitated use of services. Natural capital in terms of ownership of livestock and land was not mentioned as an important factor during the focus group discussions but was identified with being wealthier during the community meetings and was implicitly linked to having more money. The least poor identified that bad (feeder) roads during bad weather as well as the lack of ambulances hinder access to health services but they had the means to overcome these. In particular, proximity to the main tarmac road was reported as a facilitating factor to use of services. The least poor reported that they were able to take advantage of this situation even late into the night because of the good security situation.

'If one has transport we can go to bigger hospitals because there is security in the area and one can even travel at 2 am in the morning. Proximity to the main road is a facilitating factor as well as security even late into the night if the health care is available' (Least Poor, Nawangisa).

However even the least poor occasionally found it problematic to access health services when cash was unavailable.

'*What makes it easy for us to use health services is that we have some money. However, during the dry season we have no money and that makes it hard to access services' *(FGD Least Poor, Kakongoka).

The medium wealth category and poorest categories reported a lack of financial resources to meet health care costs as an outstanding barrier as one group member remarked.

*'Money is everything and yet we don't have it. If you don't have money it means no services. Don't waste your time. Just go home and die' *(FGD Medium wealth category, Kakongoka).

#### Human resources/Health literacy

The level of education and/or health literacy did not directly limit use but reportedly influenced the potential benefit from use of health care services.

*'Uneducated people can't read instructions, prescriptions or have a better understanding of health related issues. Those who are more educated are more confident, they also follow prescriptions and know why and what to do. Those of us who never went to school sometimes fail to explain our illness to the doctors because we do not know English' *(FGD Poorest, Namundudi).

#### Social resources

Social resources from friends and family were identified as useful in overcoming some of the existing barriers to utilization of health services. These resources included financial support from friends and/or family to overcome high cost of the health services; transport to attend comprehensive health services; information on where certain health services were found; and relationships with health workers that helped to make services more receptive. Social standing was assessed in terms of how one is regarded in the community. It was a probing question related to discrimination and whether access to social resources helped one to overcome this potential barrier to health care utilization. Social standing or what people think of you was not identified as a hindrance to utilizing health care services as noted below:

*'Social standing in society or what people think of you is not a hindrance to accessing health care; the biggest hindrance is money' *(FGD Poorest, Nawangisa).

The FGDs of the medium wealth category and poorest reported that social resources to use health services could be accessed through friends, relatives and employers but these were limited in terms of the potential to overcome barriers to use of services.

*'Us we do not have friends who can help us access health services when we don't have money. We don't have such people more especially we have spent the money on taking our children to school. We don't have people with big jobs in government. Friends only give advice but not money. Friends may give advice on which doctor or traditional healer to visit but no money. Friends or relatives may get you an herb or even direct you on how to prepare a treatment concoction but not give you actual money' *(Poorest, Namundudi).

The least poor on the other hand reported networks that are more useful:

*'If friends and relatives hear of your calamity they come and fetch you and take you to hospital' *(Least Poor, Kakongoka).

Knowing someone at a public health facility could be useful in terms of accessing health care services. Such a scenario is demonstrated in the following quote:

*'We had a sick child (anaemic), I needed blood urgently but was failing then all of a sudden I run into someone I know who works there so I explained and in an instant, I got all the services I needed' *(Poorest, Namundudi).

The poorest wealth category identified potential networks that could be useful in mobilizing resources to enable them use health care services. In addition they identified potential reasons for why these networks were not accessed by people in their category. For one it was perceived that helpfulness in the community was limited and was related to the ability to give something back in return.

*'No we do not have friends who help us to access health care. Maybe our MPs could have helped but they are not useful in this matter. People know that if they help you, you may not pay back' *(Poorest Category, Nawangisa).

Furthermore, it was felt that community helpfulness had also declined.

*'In those days Moslems used to help each other, it doesn't happen these days, not even during Ramadan – you can sit next to each other, one drinking porridge and the other with nothing to take. It doesn't mean anything anymore' *(Poorest, Nawangisa).

## Discussion

Barriers to healthcare utilization were reported for all the wealth categories along three different axes including: the health seeking process; health services delivery; and the ownership of livelihood assets regardless of wealth category. Income source, ownership of means of transport, and health literacy were reported as centrally useful in facilitating use of healthcare utilization for the 'least poor' and 'medium' wealth categories. However, the lack of these was a barrier for the poorest wealth category. The 'poorest' wealth category were keen to utilize public health services which are provided free of charge. However, there were perceptions that public health facilities offer low quality care with chronic gaps such as shortages of essential supplies. This study suggests that in addition to income, physical resources and free public health services, social resources were perceived as important in overcoming some of the existing barriers. However, there was variability in the extent and usefulness of social networks among wealth categories. For instance, the 'least poor category' reported the most useful networks.

Health seeking and service barriers to utilization identified in this study are consistent with those in an extensive systematic review that documented barriers to healthcare use in Uganda [37]. Other literature shows that the differential distribution of financial resources, transport and levels of health literacy disfavors the poorest and negatively influences utilization of health services [14-16,35]. Several of these barriers are recognized by policy makers and appropriate strategies have been introduced to address them. For instance the universal primary education strategy targeted at increasing school enrolment rates [44] directly impacts on the potential to improve health literacy. Similarly, the removal of user fees was in response to evidence that highlighted cost as a barrier particularly for the poor [34]. However, Xu et al concluded that while the removal of user fees did increase utilization of public health services this effect would be lost if benefits financed from the fees were not catered for. The finding that unofficial fees are sometimes demanded as a pre-requisite to service use suggests that the initial beneficial effects for the removal of user fees may have been eroded by now as predicted [34].

The availability of social resources and the use to which they were put is in line with the concept of social capital, which has been typically conceptualized as "the resources embedded in a social structure that are accessed and/or mobilized in purposive actions within a network" [45]. In this case, the resources include information, relationships, finances and transport whilst the action is the utilization of health services. That the least poor have more useful networks that can be accessed to overcome barriers to utilization of healthcare services is indicative of the variation of social capital across wealth categories. This social patterning of relationships illustrates a scenario that either contributes to or partially explains the persisting inequities in the utilization of healthcare services. It is also important to note that the level of poverty experienced in this setting seems to override the fundamental social-cultural glue of the society, necessary for enhancing the mobilization of useful social resources to overcome utilization barriers. Several studies show that having social resources can compensate for the lack of human and material capital in the process of seeking health care [20,32,33,42]. However, these studies have not taken account of how social resources are distributed amongst different wealth status groups. This study shows that the poorest who lack human and material capital also lack social capital and were not able to use social capital to overcome utilization barriers. It is perhaps then not surprising that the types of social actions described in Ivory Coast [32] are not demonstrated in this setting.

Further understanding of the role of social capital in context of healthcare utilization calls for an evaluation of the types of relationships and their density as well as the structure at community and individual level in terms of which relationships are most useful in overcoming existing barriers to healthcare utilization [46]. Future studies need to describe the types of social relations and how these influence resource mobilization to overcome existing utilization barriers.

The inequitable distribution of resources that support health care utilization and how these influence patterns of utilization supports the continued need to focus on measures that remove these disparities. In particular health system barriers such as poor health worker attitudes and practices need to be tackled to improve the quality of services since these are critical in enhancing utilization for the poorest. Poor social infrastructure illustrated by a weak public transport system affirms the necessity of tackling barriers to healthcare utilization multi-sectorally. The policy relevance of understanding how social capital relates to healthcare services utilization in this context is at least twofold. The Government of Uganda has undertaken a number of interventions to improve utilization and access to healthcare. The planned institutionalization of village health teams as part of these interventions is intended to promote community accountability mechanisms and thus scale up the appropriate use of facility-based healthcare services particularly for the poor and most vulnerable [11]. It thus serves as a deliberate measure that increases the chances of the poor to gain access to much needed health services [47]. Implicitly the success of village health teams assumes high levels of cooperation and mutually beneficial behavior at community level [48] which in turn are dependent on social capital factors such as interpersonal trust and reciprocity norms [27]. At the community level, the different levels of social capital can influence the performance of community accountability structures such as village health teams in terms of promoting utilization of healthcare services. At the individual/household level, knowing the levels of social capital could predict which individuals/households are better positioned to overcome health barriers. In particular, this can aid the design of information, education and communication (IEC) interventions to influence behavior change related to the utilization of healthcare services.

### Study limitations

Wealth ranking using community participatory methods may not be accurate since households may conceal consumption information from neighbours [23] thus potentially creating misclassification. The research team triangulated information on wealth ranking during the wider village meetings, the FGD and information from the local council officials. Information obtained may thus suffice for the purposes of this study, which was not about determining an accurate classification of wealth. Rather it was to explore perceptions on barriers to health care utilization as well as facilitating factors within homogenous wealth groups.

The study uses heterogeneous groups as it does not stratify by male/female therefore missing out on gender perspectives from separate groups. Also, there was limited representation of women in terms of numbers with for instance only one woman in the poorest wealth category in Nawangisa (see table 1). A gender perspective to barriers and facilitating factors can generate a wealth of information and it is possible that women's views were not exhaustively explored. We did however ask about any perceptions on differences in use by male and female as perceived by men and women together. From our observations, this turned out to be very useful since healthcare utilization at the family and community levels affects both male and female.

## Conclusion

Results in this study show that in addition to individual material resources and the availability of free public healthcare services, social resources are perceived as important in overcoming utilization barriers. However, there are indications that having access to social resources may compensate for the lack of material resources in relation to use of health care services mainly for the least poor wealth category. Thus, differential patterning of social resources may explain or contribute to the persisting inequities in health care utilization. Additional research using quantitative analytical methods is needed to test the robustness of the contribution of social resources to the utilization of and access to healthcare services. Such research should also analyze different types of social resources and address which ones are important in overcoming the existing barriers to accessing health care services.

## Competing interests

The authors declare that they have no competing interests.

## Authors' contributions

SKB participated in conception and design of the study, analysis and interpretation of the data, drafting the paper and revising it critically for substantial intellectual content. SPW, SG, AS, SP and GWP participated in the conception and design of the study, drafting the paper and revising it critically for intellectual content. All authors contributed to and approved the final manuscript.

## Appendix 1 – Discussion guide for the wealth ranking meetings

The wealth categories were determined using a modification of poverty ranking as described in Theis and Grady 1991. The main questions that guided discussion on wealth ranking focused on perceptions of differentiating socioeconomic characteristics. These are summarized below:

1. When we look at ourselves in this meeting, are we all the same?

2. What makes us different from one another?

3. What are the different livelihood activities in this community that differentiate us from one another?

4. What are the characteristics of those livelihood groups?

Three broad categories were identified – '*abagaiga' *(the rich), *'abafuni' *(Medium wealth category Wealth Category) and '*abaavu' *(the poorest). Ranking was mainly based on ownership of assets, type of work and lifestyle (see Table 1).

## Appendix 2 – Guiding questions for focus group discussions

### Barriers to healthcare services

The guiding questions explored for which local community factors were in general barriers to healthcare service access and utilization. The groups were encouraged to identify weaknesses in the healthcare arrangement that made it difficult to access healthcare services.

### Facilitating Factors for barriers to healthcare services

The guiding questions that explored for which local community factors were in general facilitating factors for barriers to healthcare service access and utilization. Specifically the questions probed for community perspectives on the well documented factors including:

Type of illness, acute/chronic;

Type of service sought – preventive/curative;

Behavioral characteristics: knowledge, attitudes and practices

Socio-demographic characteristics: age; sex; place of residence; occupation/means of livelihood; disabilities; distance; distance from health

The guiding questions sought to understand the relevance of less documented factors such as relationships and how they influence utilization of healthcare services e.g. 'who one knows'; 'having friends and relatives'; 'how one is regarded in the community'.

## Note

The minimum health care package encompasses a range of health promoting, preventive, curative and rehabilitative services covering conditions that contribute most to the burden of illness
